# A Complex Genome-MicroRNA Interplay in Human Mitochondria

**DOI:** 10.1155/2015/206382

**Published:** 2015-01-28

**Authors:** Santosh Shinde, Utpal Bhadra

**Affiliations:** Functional Genomics and Gene Silencing Group, Centre for Cellular and Molecular Biology, Hyderabad 500007, AP, India

## Abstract

Small noncoding regulatory RNA exist in wide spectrum of organisms ranging from prokaryote bacteria to humans. In human, a systematic search for noncoding RNA is mainly limited to the nuclear and cytosolic compartments. To investigate whether endogenous small regulatory RNA are present in cell organelles, human mitochondrial genome was also explored for prediction of precursor microRNA (pre-miRNA) and mature miRNA (miRNA) sequences. Six novel miRNA were predicted from the organelle genome by bioinformatics analysis. The structures are conserved in other five mammals including chimp, orangutan, mouse, rat, and rhesus genome. Experimentally, six human miRNA are well accumulated or deposited in human mitochondria. Three of them are expressed less prominently in Northern analysis. To ascertain their presence in human skeletal muscles, total RNA was extracted from enriched mitochondria by an immunomagnetic method. The expression of six novel pre-miRNA and miRNA was confirmed by Northern blot analysis; however, low level of remaining miRNA was found by sensitive Northern analysis. Their presence is further confirmed by real time RT-PCR. The six miRNA find their multiple targets throughout the human genome in three different types of software. The luciferase assay was used to confirm that MT-RNR2 gene was the potential target of hsa-miR-mit3 and hsa-miR-mit4.

## 1. Introduction

Mitochondria are known as the powerhouse of the cell; they are membrane bound organelles present in most eukaryotic cells. Most of the chemical reactions required in cellular respiration take place in the mitochondria. The numbers of mitochondria are generally varied from cell to cell and organism to organism. Mitochondria have their own genome (mitochondrial DNA, mtDNA) that is different from nuclear genome. Plant mitochondria genomes are relatively larger in size (180–2400 kb). In contrast, animal mitochondria harbor much smaller, highly compact, and gene dense genome. For instance, human mitochondrial genome carries 37 genes in a 16.5 kb small circular genome. Of these 13 specific protein products especially the subunit of the respiratory gene complex and the remaining 24 encode RNA products. However, there are more than 1000 proteins that have been identified in the mitochondria; they are mainly imported through the outer mitochondrial membrane by the translocase mitochondria complex enzyme or the oxidative folding pathway of the intermembrane space. Mitochondria genome imported 22 tRNA and is involved in the trafficking of different proteins through distinct molecular mechanisms [1].

Mitochondrial DNA have two strands, one is guanine-rich which is known as heavy (H) strand and the other is a cytosine-rich light (L) strand. Only noncoding segment of mtDNA is the displacement loop (D-loop), a region of 1121 bp. The heavy strand has 12 of the 13 polypeptide-encoding genes which also contain 14 of the 22 tRNA-encoding genes and both rRNA-encoding genes [1, 2]. It is found that the mtDNA evolves 6 to 17 times faster than comparable nuclear DNA gene sequences, which leads to multiple restriction fragment length polymorphisms (RFLPs) in mtDNA [3, 4]. Maternal inheritance of mtDNA has also been observed in most of the organisms including human and diseases are also resulting from base substitutions which are generally maternally transmitted [5, 6]. These mutations can be due to altered polypeptide genes, mis-sense mutations, or structural RNA, protein synthesis mutations, insertion-deletion mutations, and so forth [7]. mtDNA mutation associated with degenerative diseases involves the central nervous system such as Parkinson's disease, heart, muscle, endocrine system, kidney, and liver [8–11]. These may cause discrete clinical syndrome, such as the Kearns-Sayre syndrome (KSS), mitochondrial encephalomyopathy with lactic acidosis and stroke-like episodes (MELAS), chronic progressive external ophthalmoplegia (CPEO), myoclonic epilepsy with ragged-red fibers (MERRF), and neurogenic weakness [12].

In earlier studies, it has been shown that microRNA (miRNA) were identified in human mitochondria [13–21]. These cell organelles play a vital cellular function, which is required in the fine-tuning of the posttranscriptional regulation by an alternative pathway [13]. It was anticipated that few miRNA could be imported and/or processed in the mitochondria for posttranscriptional silencing of mitochondria as well as nuclear protein imported to the mitochondria [21, 22]. The existence of miRNA in the mitochondria suggests that these miRNA may be processed outside and enter into the mitochondria to control the mitochondrial genes related to different diseases [18]. After predicting pre-miRNA and miRNA candidates by the in silico analysis, we also verified the existence of pre-miRNA and mature miRNA candidate of human mitochondrial skeletal cells using quantitative RT-PCR and Northern analysis. The variable expression of six mitochondrial miRNA (mt-miRNA) was noticed in highly purified mitochondrial fraction and finally we estimated their potential target inside the mitochondrial genome on difference by in silico analysis in controlling different disease regulated genes.

## 2. Materials and Methods

**Figure 1:**
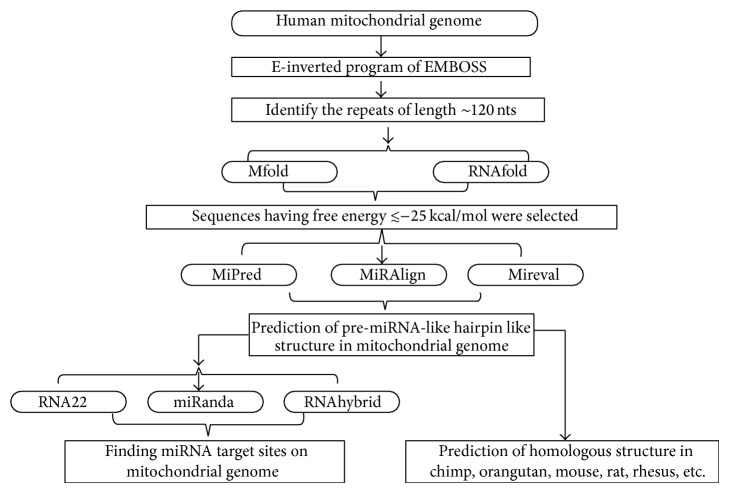
Flow chart of miRNA prediction in mitochondrial genome (bioinformatics analysis).

In this study we have predicted pre-miRNA and mature miRNA and their identified targets in the mitochondrial genome. We have also constructed an interaction map that unravels the relationship between miRNA and mitochondrial genome. A complete flow chart of this study (bioinformatics analysis) is shown in Figure 1.

### 2.1. Finding Inverted Repeats

The complete genome of* Homo sapiens* mitochondrion was downloaded from NCBI (http://www.ncbi.nlm.nih.gov/) as a query NC-012920.1, which is 16,569 nt in length. The locations of mitochondrial genes (mtDNA) and functions are collected from MITOMAP, a human mitochondrial genome database (http://www.mitomap.org).

This complete genome sequence of* Homo sapiens* mitochondrion is screened using EMBOSS e-inverted program (http://emboss.bioinformatics.nl/cgi-bin/emboss/einverted) to identify the repeats of length ~120 nucleotides that are capable of forming hairpin stem-loop structures. The following parameters are used for analysis: gap penalty: 12, minimum score threshold: 10, match score: 3, mismatch score: −4, and maximum extent of repeats: 500 bp.

### 2.2. Prediction of Pre-miRNA-like Hairpin Structures

Total 110 sequences were used as an input for Mfold and RNAfold program for identification of hairpin like structures.

#### 2.2.1. RNAfold

RNAfold program (http://rna.tbi.univie.ac.at/cgi-bin/RNAfold.cgi) is maintained by Vienna RNA web server and used for prediction of minimum free energy structures and base pair probabilities from single RNA or DNA sequences. The RNAfold web server predicts secondary structures of single stranded RNA or DNA sequences. Default parameters were used for prediction of the miRNA.

#### 2.2.2. Mfold

Mfold program available at http://mfold.rna.albany.edu/?q=mfold is used for prediction of secondary structures. It gives detailed output in the form of structure plots, single strand frequency plots, and energy dot plots for folding of single stranded sequences.

### 2.3. miRNA Prediction

Total 110 miRNA candidate sequences were scanned to find out real versus false miRNA. Three types of software were used for proper identification of miRNA. These types of software are MiPred, MiRAlign, and Mireval.

*MiPred.* The MiPred web server (http://www.bioinf.seu.edu.cn/miRNA/) was used to distinguish the real pre-miRNA from other hairpin sequences with similar stem-loops (pseudo pre-miRNA); a hybrid feature that consists of local contiguous structure-sequence composition, minimum of free energy (MFE) of the secondary structure, and *P* value for randomization test was used. Besides, a novel machine-learning algorithm, random forest (RF), was introduced. Analysis of the predicted sequence, MiPred, decides whether it is a real pre-miRNA-like hairpin sequence [24].

*MiRAlign.* This software was used as a novel genome-wide computational approach to detect miRNA in animals based on both sequence and structure alignment; it has higher sensitivity and comparable specificity than other reported homologue searching methods. miRAlign follows two approaches; first, to be able to find distant homologs, miRAlign requires neither the sequence conservation of the whole pre-miRNA sequence nor the nearly perfect match of the 22 nt mature part but just assumes relatively loose conservation of the mature miRNA sequence. Secondly, more properties of miRNA structure conservation are considered. miRAlign introduces a structure alignment strategy and each single miRNA can be used as a query to do homology search [25].

*Mireval.* This software works in three steps. In the first step it finds miRNA precursor-like structure, in the second step it searches for sequence similarity with a known miRNA, and in the third step it searches for the sequence conservation. Input sequence will be folded and evaluated by an SVM classifier (triplet-SVM) and then blasted against the miRBase dataset on all organisms and significant similarities will be reported. It also reports input sequence on their corresponding genome and the conservation score of this genomic location will be downloaded from the Ensembl database [26]; this software is available at http://tagc.univ-mrs.fr/mireval/.

### 2.4. miRNA Similarity Search

All six miRNA are searched for similarity in miRBase, a microRNA repository [27]. miRBase has a single sequence search method that uses BLAST for searching similar miRNA in miRBase database. All default parameters are used for similarity search by an *E*-value cutoff of 10 and maximum number of hits of 100.

### 2.5. Calculation of Physicochemical Properties of Six miRNA

Physicochemical properties such as %GC content, %AU content, molecular weight (Kilo Dalton), free energy (*δ*G in Kcal/mol), and composition were calculated by employing Oligo Calc: Oligonucleotide Properties Calculator [28].

Molecular weight and free energy were calculated by the following formula:

(1) Molecular  weight  Mw =An×329.21+Un×306.17  +Cn×305.18+Gn×329.21+159.0,

where *An*, *Un*, *Cn*, and *Gn* are the number of respective nucleotides of the RNA molecule under consideration. Additionally, weight 159.0 gm/mole is added that accounts to 5'triphospahte:

(2) Free  energy  δG: δG=RT ln⁡RNA·  templateRNAtemplate.

Both (1) and (2) assume that the annealing occurs under the standard conditions of 50 nM primer, 50 mM Na+, and pH 7.0.

### 2.6. Homology Search

Homology search was carried out with six Human miRNA predicted against chimp, orangutan, mouse, rat, and rhesus genomes using BLAT.

#### 2.6.1. BLAT

This is free online software (University of California, Santa Cruz Genome Browser, UCSC Genome Browser) and BLAT can be found at http://genome.ucsc.edu/cgi-bin/hgBlat?command=start [29, 30]. BLAT on DNA is designed to quickly find sequences of 95% and greater similarity of length of 20 bases or more. It may miss more divergent or shorter sequence alignments. It will find perfect sequence matches of 25 bases and sometimes find them down to 20 bases. BLAT is not BLAST. DNA BLAT works by keeping an index of the entire genome in memory. The index consists of all nonoverlapping 11-mers except for those heavily involved in repeats [29].

### 2.7. Isolation of Human Mitochondria

Human skeletal muscle myoblast cells (HSMN Lonza) were cultured in tissue culture flask at a seeding density of 3,500–4000 cells/cm at 37°C with 5% CO_2_. The growth media were supplemented with specific growth factor: rhEGF, 0.5 mL; dexamethasone, 10 mL; FBS, 50 mL; and GA-1000, 0.5 mL (SkBM-2 Basal Medium and aq. Single Quot, Lonza). At 50–70% confluency myotube differentiation was induced by changing the media and growing cells in DMEM supplemented with only 2% of horse serum for 3–5 days until the myotubes were visible. The media were changed every day with fresh media; the myotubes were collected by trypsinization and centrifuged at 1000 ×g for 10 minutes.

To isolate mitochondria human skeletal muscle myoblast cells were centrifuged at approximately 370 g for 10 minutes. Cells were resuspended in NKM buffer (1 mM TrisHCl, pH 7.4, 0.13 M NaCl, 5 mM KCl, and 7.5 mM MgCl_2_). The supernatant was decanted and the cells were pelleted; this step was repeated for two times. Then cells were resuspended in homogenization buffer (10 mM Tris-HCl, pH 6.7, 10 mM KCl, 0.15 mM MgCl_2_, 1 mM PMSF, and 1 mM DTT). Cells were homogenized and incubated for 10 minutes on ice. This homogenate is then centrifuged with 2 M sucrose solution at 1200 g for 5 min and supernatant is transferred to another tube. This step is repeated twice by transferring the supernatant to a new tube each time and discarding the pellet. Then centrifugation is carried out at 7000 ×g for 10 minutes. Mitochondrial pellet was resuspended in mitochondrial suspension buffer (10 mM TrisHCl, pH 6.7, 0.15 mM MgCl_2_, 0.25 M sucrose, 1 mM PMSF, and 1 mM DTT). Recentrifugation is carried out to repellet the mitochondria at 9500 ×g for 5 minutes.

### 2.8. Mitochondrial RNA Extraction and Quantification

Mitochondria were resuspended in 100 mL of lysine solution; a population of mitochondria enriched human myoblast cell lysate was collected and washed with PBS and used for RNA isolation. Total cellular RNA was isolated by Trizol reagent (Invitrogen) and treated with RNase-free DNase I (Promega) for 1 h at 37°C. The total RNA was quantified with spectrophotometer, Nanodrop ND-1000, to assay the degree of enrichment of mitochondrial RNA and the risk of cytoplasmic contamination at the mitochondrial RNA and DNA level. miRNA were isolated from mitochondrial fraction by using miRNeasy kit (Qiagen, Valencia, CA), according to the manufacturer's protocol.

### 2.9. Western Blot Hybridization

Human skeletal muscle cells and enriched mitochondrial skeletal cell pellets were lysed with ice-cold RIPA buffer containing protease inhibitor. After centrifuging at 12,000 rpm for 10 min at 4°C, protein was collected from supernatant and quantified by Bradford method (BIO-RAD) using multimode reader. 100 *µ*g of human cytosolic and enriched mitochondrial protein was loaded for each sample and the protein was transferred to PVDF membrane (Amersham Biosciences). The membrane was blocked in TBS with 0.1% Tween 20 (TBST) containing 5% nonfat dry milk for 1 hr. Then the membrane was incubated with primary antibody and kept at 4°C overnight. Membrane was washed with TBST for three times for 15 min and incubated with corresponding horseradish peroxidase (HRP) conjugated secondary antibody (1 : 2000; Santa Cruz) for 1 hr. Again TBST wash was given for 15 min and the blots were visualized with chemiluminescent reagents.

**Figure 2:**
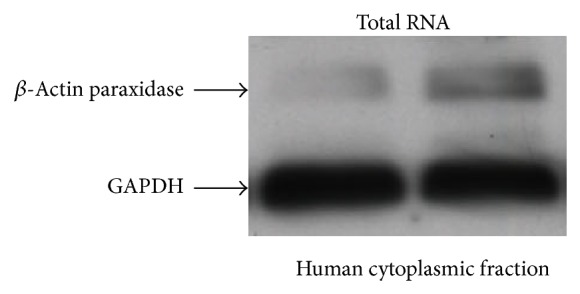
Blot showing quantitative Western blot analysis of *β*-actin peroxidase in relation to loading differences of GAPDH probes extracted from whole cell total RNA.

The amount of protein was analyzed by Western blot using *β*-actin peroxidase (Sigma ref. A3854) and rabbit GAPDH. The Western blot protocol and the secondary antibodies used are described earlier (*β*-actin peroxidase 1 : 1000 dilution, GAPDH 1 : 5000 dilution) (Figure 2).

### 2.10. RT-qPCR

**Figure 3:**
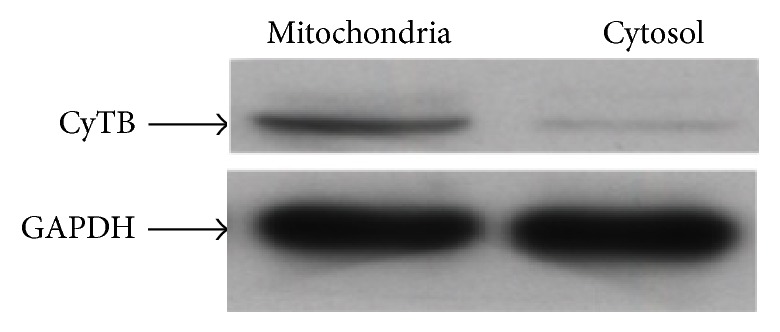
Blots showing mitochondrial probe CyTB in enriched mitochondrial and cytosolic RNA loading in two different lanes of the gel. The CyTB differential expression is compared to GAPDH loading.

To check quality and purity of mitochondrial fractionation, mitochondrial gene (CYTB) and nuclear gene (GAPDH) were used to measure both transcripts expression and DNA relative quantification (Figure 3). Reverse transcription was carried out and then real time quantitative PCR (RT-qPCR) with Roche Light Cycler 480. To check the mitochondria enrichment and genomic contamination, DNA amplicons of the same mitochondrial genes and nuclear genes were obtained using the same primers. The amplicon concentrations were measured by RT-qPCR. GAPDH was used to normalize the CT data and also used as a calibrator to calculate the mitochondrial to nuclear DNA ratio.

### 2.11. MicroRNA Detection

The RT-PCR analysis was performed following the Exiqon protocol; three samples of mitochondrial extract (1, 5, or 35 *µ*L of RNA) were reverse-transcribed in 40 *µ*L reaction and diluted 100-fold in the RT-PCR analysis using miRCURY LNATM Universal RT microRNA PCR kit (Exiqon). Negative control excluding enzymes from reverse transcription reactions was performed and profiled like the samples. Each PCR panel contains a PCR control and 3 PCR sets for reference gene. CP and melting curve analysis was done by using the Roche Light Cycler 480 software.

### 2.12. Quantitative Northern Blot Analysis

To estimate the human mitochondria mRNA level, total mitochondria rich human myoblast cell extract was used. 100 *µ*L RNA was denatured, subjected to electrophoresis in agarose gels, transferred to nylon membrane, and hybridized with 6 separate human antisense pre-miRNA radio-labelled as described earlier [31]. The amount of radioactivity in each band was estimated using a Fuji phosphorimager. 5 *µ*g of DNA free RNA was added as template for cDNA synthesis using MMLV (Moloney Murine Leukemia Virus) reverse transcriptase (Promega). Further amplification of cDNA was performed by PCR using gene specific primers.

### 2.13. MicroRNA Target Identification

Six novel miRNA identified were used for miRNA target identification with help of three types of software: miRanda, RNA22, and RNAhybrid, as described here.

*miRanda.* miRanda (version 1.0b), available at http://www.microrna.org/microrna/home.do, a dynamic programming algorithm that relies on the sequence complementarity [32, 33], was employed for prediction of target sites of miRNA expressed in mitochondria. miRanda allots higher weights to matches at the 5′ end of the mature miRNA while considering the free energy of the RNA-RNA duplex [29] and the degree of conservation of the miRNA target across related genomes. The analysis was performed keeping the following cut-off values for prediction of target sites: gap open penalty 2.0, gap extend: 8.00, match score (*S*) ≥ 150.00, duplex free energy (Δ*G*) = −25.00 kcal/mol, and scaling parameter (*w*) = 3.00. The six human mitochondrial mature miRNA and complete mitochondrial genome were used as reference and query sequences, respectively, as input to miRanda. 

*RNA22.* This software predicts microRNA targets which is maintained by IBM at https://cm.jefferson.edu/data-tools-downloads/rna22-v2-0/. RNA22 does not rely upon cross-species conservation, thus allowing the discovery of microRNA binding sites that may not be present in closely related species. It first finds putative microRNA binding sites in the sequence of interest and then identifies the targeting microRNA. RNA22 can identify putative microRNA binding sites without a need to know the identity of the targeting microRNA; this permits the identification of binding sites even if the targeting microRNA is not among those currently known [34].

*RNAhybrid.* RNAhybrid is the online microRNA target prediction software available at http://bibiserv.techfak.uni-bielefeld.de/rnahybrid. It has some useful features; among these are the possibility to disallow G:U base pairs in the seed region and a seed-match speed-up, which accelerates the program by a factor of 8. The algorithmic core of RNAhybrid is a variation of the classic RNA secondary structure prediction. Instead of a single sequence that is folded back onto itself in the energetically most favorable fashion, RNAhybrid determines the most favorable hybridization site between two sequences [35].

### 2.14. Dual Luciferase Reporter Assays

Normal and mutated 3′ UTR of MT-RNR2 gene was subcloned using PCR based methods to generate the reporter vectors containing miRNA binding sites. Then this construct was inserted into the multiple cloning sites downstream of the luciferase gene in the psiCHECK-2 luciferase miRNA expression reporter vector.

For the luciferase assay, HEK-293 cells were cultured to 70–80% confluence in 24-well plates and cotransfected with psiCHECK2-MT-RNR2-3′ UTR or psiCHECK 2-mut-MT-RNR2-3-UTR vector plus 100 mM anti hsa-miR-mit3 or hsa-miR-mit4 separately using Lipofectamine 2000 (Invitrogen), according to the manufacturer's protocol. The cells were incubated with transfection reagent/DNA complex for 5 h and refreshed with fresh medium containing 10% FBS. At 48 h after transfection firefly and renilla luciferase activities were evaluated using the dual luciferase reporter assay system (Promega) and the renilla luciferase activity was normalized to firefly luciferase activity.

## 3. Results

### 3.1. Predicted Pre-miRNA and miRNA Candidates

By an experimental in silico approach, total 268 inverted repeats are generated as an output of emboss program that are used for identification of ~120-nucleotide sequence. The number reduces to 110 sequences. The screened sequences were used for input for Mfold and RNAfold program for identification of hairpin like structures of the pre-miRNA. The number was further reduced and finally 6 mature and pre-miRNA were found suitable as pre-miRNA or miRNA candidates.

**Figure 4:**
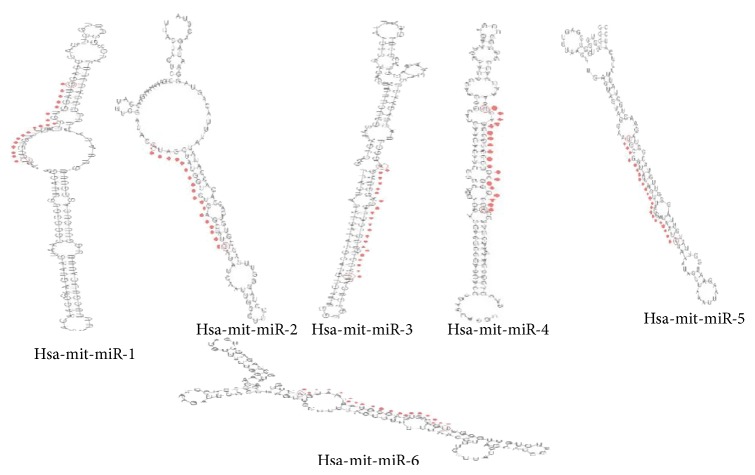
Predicted pre-miRNA and miRNA structure.

**Figure 5:**
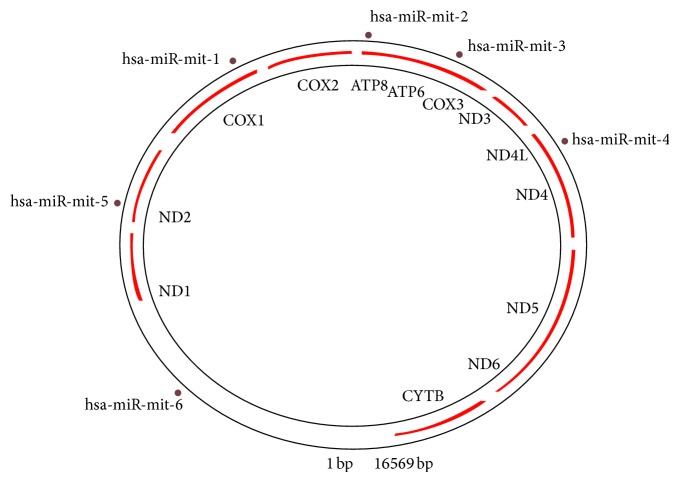
Schematic presentation of human miRNA prediction in mitochondrial genome.

Total six human precursors and mature miRNA were predicted with help of these types of software (Figures 4 and 5 and Table 1). Location of mature, pre-miRNA and mitochondrial genes in human mitochondrial circular genome is shown in Figure 4. The other details such as pre-microRNA, mature miRNA, and their genomic location on mitochondrial genome and sequences are summarized in (Tables S2 and S3) (Supplementary Data 1 in Supplementary Material available online at http://dx.doi.org/10.1155/2014/206382.).   Six miRNA and their functions were detected in controlling myoblast proliferation and differentiation. Several miRNA were detected in robust and reliable fashion in the mitochondrial RNA extracts.

### 3.2. Mitochondrial MicroRNA and Human Diseases

All six mature miRNA were used for similarity search. hsa-miR-mit-1 has shown one hit with human miRNA hsa-miR-331-5p with 59 score and 4.9 *E*-value. hsa-miR-331-5p has human tumor suppressor role in prostate cancer, leukemia, gastric cancer, liver cancer, Parkinson's disease, and so forth [36, 37]. The hsa-miR-mit-2 has three matches with let-7 human miRNA, that is, hsa-let-7i, hsa-let-7b, and hsa-let-7g. Let-7 miRNA have been predicted or experimentally confirmed in a wide range of species including human to* C. elegans.* They have various roles including developmental one to inhibit cell proliferation in breast, colon, and hepatic cancers, lymphoma, and uterine leiomyoma [38]. hsa-miR-mit-3 has two matches with hsa-miR-93 and hsa-miR-20a. These miRNA are miR-17 miRNA precursor family; these are a group of related small noncoding RNA. hsa-miR-93 and hsa-miR-20a have been found to be overexpressed in a multiple cancer types, induce cell proliferation and deletion of these miRNA clusters, in mice, are lethal, and cause lung and lymphoid cell developmental defects [39]. hsa-miR-mit-5 has three hits found, that is, hsa-let-7a, hsa-let-7f, and hsa-let-7g, whereas hsa-miR-mit-4 and hsa-miR-mit-6 do not have any similarity with human miRNA.

### 3.3. Physicochemical Properties

The size of the miRNA varied between 19 and 22 nucleotides. Physicochemical properties such as %GC content, %AU content, molecular weight (Kilo Dalton), free energy (*δ*G in Kcal/mol), and composition were calculated (Table S1) (Supplementary Data 1). %GC content of all six miRNA varies from 36% to 45% and %AU content from 55% to 64%. Molecular weight (Kilo Dalton) and free energy (*δ*G in Kcal/mol) of six miRNA are also provided in the table. Moreover alignment results show that seed sequence of few miRNAs are perfectly aligned and some aligned sequences shows one or two base pair mismatches. These pre-miRNA and mature miRNA were mapped in the intergenic region of the mitochondrial genome. The existence of the pre-miRNA and mature miRNA was experimentally evaluated by the Northern blot analysis in the mtRNA of the skeletal myoblast.

### 3.4. Enriched RNA from Isolated Mitochondria

**Figure 6:**
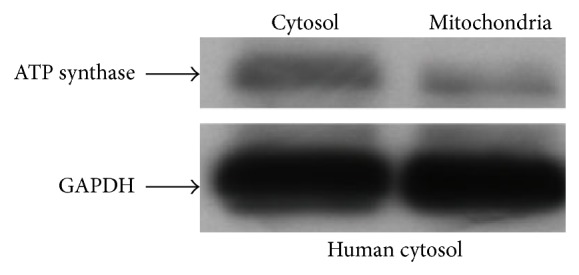
Western blot hybridization with ATP synthase isolated from mitochondria enriched and cytosolic RNA.

To evaluate the degree of mitochondrial enrichment, equal amounts of protein from whole cell extract and mitochondrial fraction were assayed by quantitative Western blot analysis. Two separate blots were hybridized with ATP synthase (mitochondrial enzyme) and cytoplasmic protein GAPDH (Figure 6). The amount was strongly depleted in the mitochondrial fraction.

**Figure 7:**
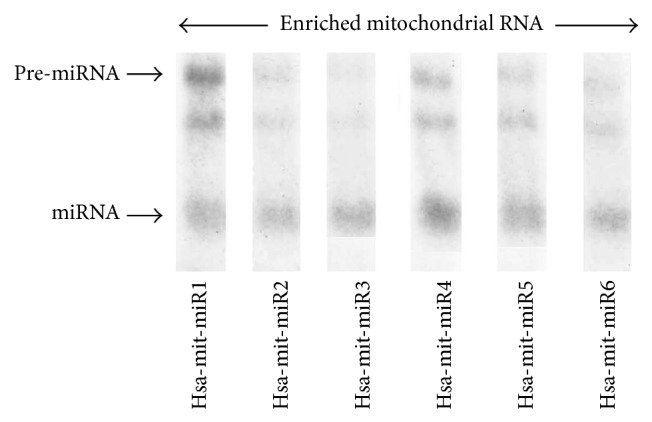
Autoradiogram showing Northern blots hybridization probed with six different pre-miRNA antisense probes. Northern blot was performed with equal amount of enriched mitochondrial RNA.

**Figure 8:**
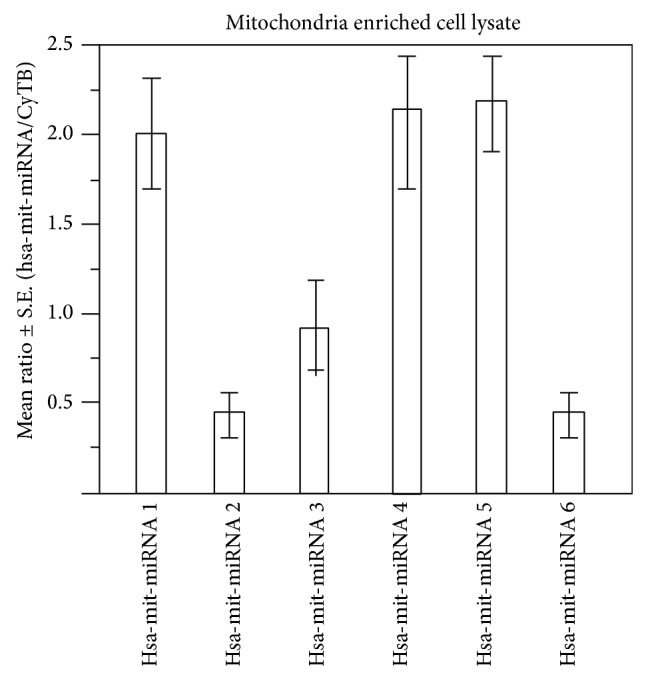
Ratio of the six miRNA in comparison to CyTB. Relative ratios in triplicate were calculated following RT-PCR. The height of the bar is proportional to the amount of human mitochondrial miRNA.

In addition, the relative ratios of mitochondrial transcripts from the CYTB gene and GAPDH transcripts were estimated by quantitative real time PCR. The mRNA level of CYTB was dramatically increased in the mitochondrial fraction in comparison to the cytoplasmic nuclear extracts. These results indicate a high abundance of mtRNA in the mitochondrial fraction which also suggest a very low level of contamination of genomic RNA in this fraction. The concentration of small noncoding RNA was measured relative to the human miRNA control lane. A lower level of expression in mitochondrial miRNA relative to human miRNA suggested that miRNA concentration is lower in mitochondria, but a significant amount of miRNA was also enriched in the mitochondrial fraction (Figure 7). Relative estimation of miRNA was conducted by the real time PCR (Figure 8).

The amount of six predicted miRNA and their precursors in human mitochondria was assayed by quantitative Northern analysis (Figure 6).

The hybridization was conducted by probing with labeled predicted sequences. The mtRNA extracted from purified mitochondria appeared to be reliable and good source for miRNA profiling. The separate Northern blots with equal amount of mitochondrial RNA were subjected to electrophoresis and hybridized separately with six miRNA probes. The Northern blots were reprobed with 5S RNA for gel loading of each Northern blot. Three miRNA were found in considerable trace level, whereas the other three miRNA are in detected level, suggesting that expression level of three miRNA reaches to the minimum detectable level when equal level of RNA (50 *μ*g) was used for each lane.

We also found a longer precursor detected from the same probe suggesting that longer pre-miRNA as well as mature microRNA anticipated that miRNA showed variable level of expression in the mitochondria. To determine whether mt-miRNA were expressed in low level, we have employed RT-PCR assay to estimate trace amount of miRNA. All six miRNA assays lead to positive results.

### 3.5. MicroRNA Evolutionary Trend

Total six human mature miRNA are used for homology search against chimp, orangutan, mouse, rat, and rhesus genomes. In chimp all six human miRNA are found to be showing homology with chimp mitochondrial genome. It conceives that no new mitochondrial miRNA evolved during evolution from chimp to human. Human mt-miRNA may be evolved before chimp. Therefore human mt-miRNA is quite old and evolved before chimp. We got six chimp pre- and mature miRNA in chimp genome. Next we have searched homology in orangutan genome; here we found that total two miRNA, namely, hsa-miR-mit-1 and hsa-miR-mit-3, are showing similarity with orangutan genome. In mouse and rat genome we got a single homology with hsa-miR-mit-6 miRNA, whereas in rhesus genome we have not found any single homology structure with human miRNA. Taken together these results suggest that, among the human mt-miRNA, hsa-miR-mit-6 is the most primitively synthesized as they both are present from mouse or rat to human. Total 10 homologous miRNA are found in five organisms' sequences. The details of all predicted miRNA such as location binding sites start and end are given in Table 1.

### 3.6. Intersection of miRanda, RNA22, and RNAhybrid Software

Total 229 miRNA target sites were predicted by all three types of software, namely, miRanda, RNA22, and RNAhybrid. miRanda has predicted 126 miRNA targets whereas RNA22 and RNAhybrid have predicted 97 and 6 miRNA targets, respectively. More target sites were predicted by RNA22 and miRanda whereas RNAhybrid has predicted only 6 miRNA targets.

**Figure 9:**
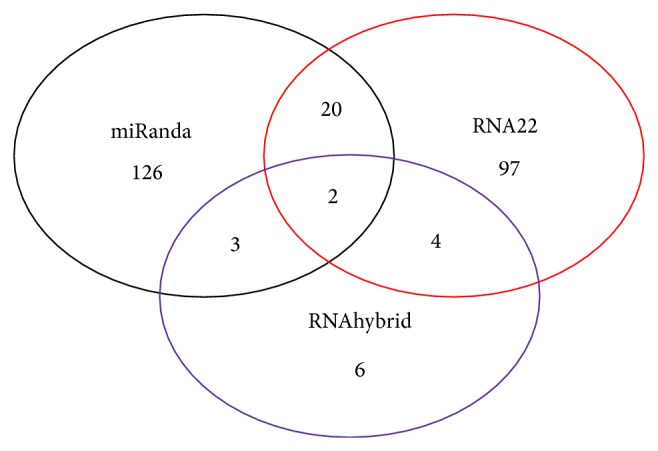
Intersection of miRanda, PicTar, and TargetScan results.

**Figure 10:**
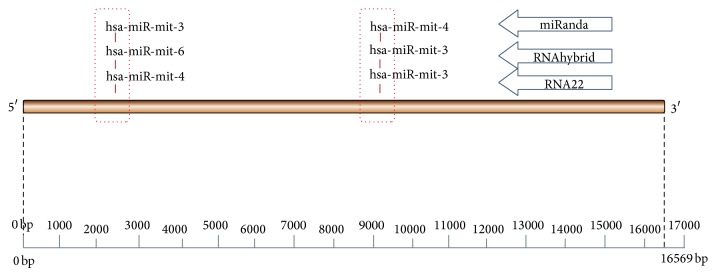
Schematic representation of common miRNA target sites on mitochondrial genome.

All 229 miRNA targets were analyzed for intersection, which is shown in Figure 8. It shows Miranda has 126 targets and RNA22 has 97 targets, intersections results of these softwares shows that 20 common miRNA target sites are sharing similar binding sites. In RNA22 and RNAhybrid 4 common target sites are there, whereas in RNAhybrid and miRanda 3 common binding sites were found. Intersection of all three types of software shows that 2 miRNA binding sites shared common binding sites as shown in Figure 9. Three miRNA found to be involved in two binding sites are hsa-miR-mit3, hsa-miR-mit4, and hsa-miR-mit6. They are binding in region between 2403-2446 (hsa-miR-mit3, hsa-miR-mit6, and hsa-miR-mit4) and 9184-9222 (hsa-miR-mit3, hsa-miR-mit3, and hsa-miR-mit4), respectively (Figure 10).

The mitochondrial genome contains 13 protein-coding genes. Many of these genes encode the transport chain. Mitochondrial rRNA is encoded by MT-RNR1 (12S) and MT-RNR2 (16S). The binding region between 1671 and 3229 is the region for MT-RNR2 gene (16S), where three miRNA, hsa-miR-mit3, hsa-miR-mit6, and hsa-miR-mit4, are binding and making a cluster at 2403-2446 binding sites.

Hence, these three miRNA may have role in regulating the activity of MT-RNR2 gene. MT-RNR2 gene (mitochondria encoded 16S RNA) encodes a mitochondrial ribosomal RNA (reran) in humans. The MT-RNR2 gene also encodes the Humanin polypeptide that has been the target of Alzheimer's disease research [40, 41], so these miRNA might be the regulators in Alzheimer's disease.

Second miRNA cluster (hsa-miR-mit3, hsa-miR-mit3, and hsa-miR-mit4) is binding at 9184-9222 site which is the site for two genes, namely, MT-ATP6 (ATPase6∖ATP synthase F0 subunit 6) which lies in region 8527-9207 and second gene MT-CO3 (COIII∖cytochrome C oxidase subunit III) which lies in region between 9207 and 9990. MT-ATP6 is an ATP synthase. (The official name of this gene is “mitochondrially encoded ATP synthase 6.”) It forms one part (subunit) of a large enzyme called ATP synthase. This enzyme, which is also known as complex V, is responsible for the final step of oxidative phosphorylation. It is found that mutations in the MT-ATP6 gene result in neuropathy, ataxia, and retinitis pigmentosa, which lead to muscle weakness, vision loss, and the other features. Cytochrome C oxidase is a unit of three complexes; cytochrome C oxidase subunit 3 is an enzyme that in humans is encoded by the MT-CO3 gene. Defects in MT-CO3 are a cause of Leber hereditary optic neuropathy (LHON). LHON is a maternally inherited disease resulting in acute or subacute loss of central vision, due to optic nerve dysfunction. Cardiac conduction defects and neurological defects have also been described in some patients. These three miRNA (hsa-miR-mit3, hsa-miR-mit3, and hsa-miR-mit4) may have role in regulating activity in neuropathy, ataxia, retinitis pigmentosa, and LHON.

### 3.7. miRanda versus RNA22

**Figure 11:**
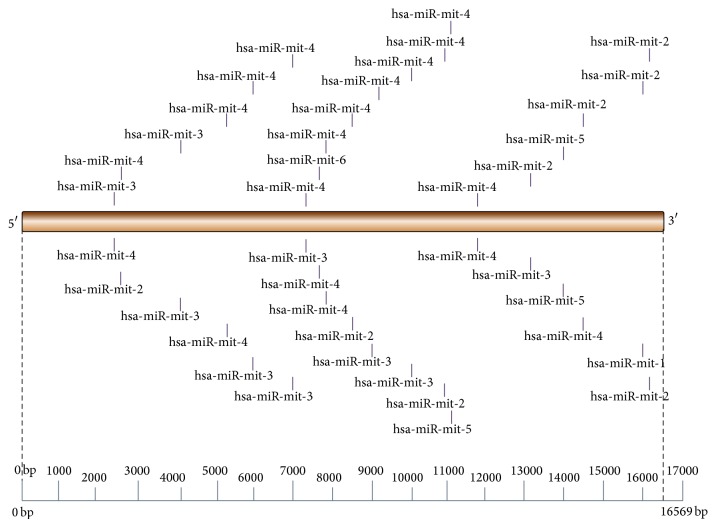
Schematic representation of miRNA conserved target on mitochondria (miRanda versus RNA22).

Comparison of miRanda and RNA22 has found that 20 miRNA targets share binding site similarity with each other. These 20 miRNA are mapped on mitochondrial genome with respect to their locations (Figure 11); they cover length around 2408 to 16302 on mitochondrial genome. They regulate almost every gene on entire mitochondrial genome except genes present in region in between 0 nucleotides and 2407 nucleotides.

### 3.8. Interaction Map

**Figure 12:**
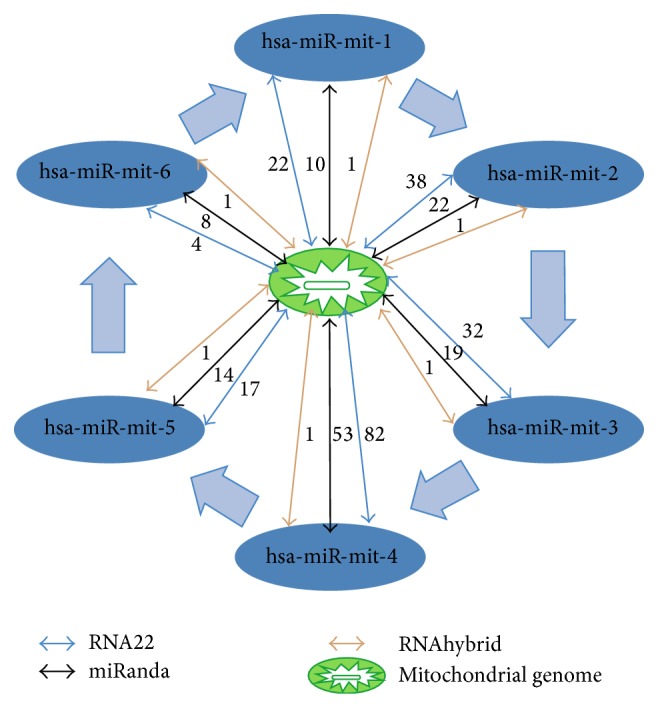
Interaction map of miRNA and mitochondrial genome. Interactions among mitochondrial genome and miRNA are depicted with arrows, number on that arrow represents number of miRNA targets predicted by the software, and different colors of arrow represent type of software.

The interaction map is plotted to show interrelation between genomic miRNA and mitochondrial genome. Total six miRNA are interacting with mitochondrial genome. This is shown with respect to output of three types of software, that is, number of miRNA predicted by the software. A typical arrow corresponds to type of software predicting the target and the number on that arrow represents number of miRNA targets predicted by the software (Figure 12).

### 3.9. miRNA Target on Mitochondrial Diseases

Total 28 diseases/disorders regions miRNA targets are identified by using miRanda and RNA22 software (Tables S4 and S5) (Supplementary Data 1), which shows miRNA binding sites in mitochondrial disease associated genes. In Table S4 28 diseases are listed with their genes. In Leber hereditary optic neuropathy (LHON) 10 genes are involved; these are MT-ND1, MT-ND4, MT-ND4L, MT-ND6, MT-CO1, MT-CO3, MT-CYB, MT-ND2, and MT-ND5. miRanda data has been explored for LHON; all genes are found to be under regulation of miRNA. In Leigh syndrome MT-ATP6, MT-ND1, MT-ND2, MT-ND3, MT-ND4, MT-ND5, MT-ND6, MT-TK, MT-TV, and MT-TW are listed, in which 8 genes are under regulation of miRNA but genes MT-TK and MT-TV do not have any miRNA targets. MELAS syndrome is also under regulation of miRNA. Gene MT-RNR2 is located on mitochondrial genome at 1671-3229 bp position and codes for 16S ribosomal RNA. The MT-RNR2 gene also encodes the Humanin polypeptide that has been the target of Alzheimer's disease research and is found to be regulated on eight sites by two miRNA, namely, hsa-miR-mit4 and hsa-miR-mit3. Diabetes mellitus and deafness are associated with abnormalities in gene MT-TL1, which codes for tRNA leucine 1. This gene is under control of hsa-miR-mit3, which is binding at 3237-3260 target sites. Six diseases are not found to be under regulation of any miRNA; see Table S4 for more details. Table S6 (Supplementary Data 1) shows information of mitochondrial genes, position, and description. miRanda and RNA22 target intersection data is also explored for miRNA target intersection in all mitochondrial diseases. In LHON out of 10 genes 7 are found to be regulated by miRNA. MT-ND4L, MT-CO3, and MT-CYB are not controlled by any miRNA. In MELAS syndrome MT-TK, MT-TV, and MT-TW are not targeted by any miRNA. In Alzheimer's disease MT-RNR2 gene is under control of hsa-miR-mit3, hsa-miR-mit2, hsa-miR-mit3, and hsa-miR-mit4. MT-ND6 a diabetes and myopathy gene which is also under influence of hsa-miR-mit4 and hsa-miR-mit2 at 14344 to 14377 bp position; see Table S5 for more details (Supplementary Data 1).

Intersection of data of all three types of software (miRanda, TargetScan, and PicTar) shows that three genes are sharing common binding sites for these miRNA. MT-RNR2 gene, which is responsible for Alzheimer's disease, is also found to be under regulation of three miRNA, that is, hsa-miR-mit3, hsa-miR-mit6, and hsa-miR-mit4. MT-ATP6 is one of the important genes which encodes ATP synthase F0 subunit 6; fluctuation of this genes activity is associated with diseases such as LHON, Leigh syndrome, neuropathy, ataxia, and retinitis pigmentosa, NARP, and bilateral necrosis of striation. hsa-miR-mit3, hsa-miR-mit3, and hsa-miR-mit4 bind at region 9184-9222 on MT-ATP6 and control all the diseases associated with them. MT-CO3, which codes for cytochrome C oxidase subunit III, is responsible for diseases such as LHON and MELAS syndrome also under regulation of these miRNA. We believe that these miRNA which are binding at the same region control activity of genes in strong manner by way of competitive selection.

### 3.10. Luciferase Assay of hsa-miR-mit3 and hsa-miR-mit4

**Figure 13:**
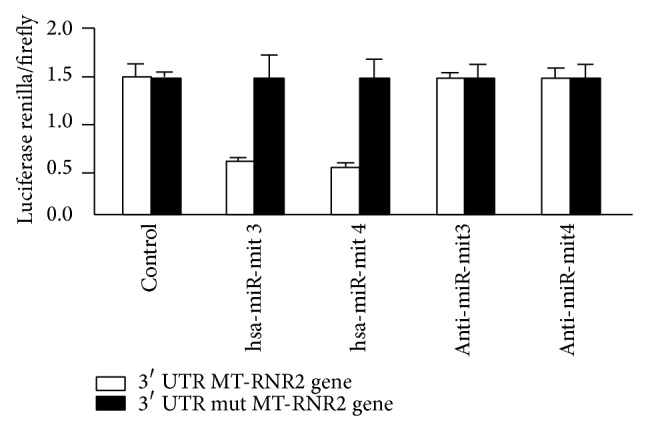
Luciferase assay of hsa-miR-mit3 and has-miR-mit4. Luciferase assay was used to invent whether MT-RNR2 was the direct target of hsa-miR-mit3 or hsa-miR-mit4 microRNA separately. A wild type and a mutated 3′ UTR of MT-RNR2 gene were subcloned into the psiCHECK-2 luciferase miRNA expression reporter vector as described earlier [23]. The transfected cells were cotransfected with 100 mM anti-hsa-miR-mit3 or the same amount of anti-hsa-miR-mit4.

A luciferase assay was performed to detect if MT-RNR2 gene (mitochondria encoded 16S RNA) was the direct target for hsa-miR-mit3 and/or hsa-miR-mit4 as described earlier [23]. The experiment data showed that the value of renilla/firefly luciferase was significantly lower in the hsa-miR-mit3 or hsa-miR-mit4 treatment cells following transfection with the 3′ UTR of the MT-RNR2 gene, whereas the value of renilla/firefly luciferase showed no difference following transfection with the mutated 3′ UTR of MT-RNR2 genes compared to the control (Figure 13). These findings clearly suggested that hsa-miR-mit3 and hsa-miR-mit4 may downregulate MT-RNR2 gene expression and that the 3′ UTR of MT-RNR2 is the possible target for hsa-miR-mit3 or hsa-mir-mit4 microRNA.

### 3.11. miRNA Recognition in the Mitochondria

Human miRNA were monitored by RT-PCR analysis of specific miRNA assays and controls. The analyses were further performed on three increasing quantities of the mitochondrial RNA input. The mtRNA extracted from purified mitochondria appeared to be a good source for miRNA profiling. The top-six most common detected miRNA in the mitochondria of the human myotubes were in homology with the following human genomic miRNA. We detected 6 miRNA known for their functions in controlling myoblastic proliferation and differentiation. The miRNA let-7 family was well represented. Many miRNA were detected in the mitochondrial RNA extract, so their potential targets in the mitochondrial genome should be investigated based on their location in the 3' end or interdomain of the gene.

**Figure 14:**
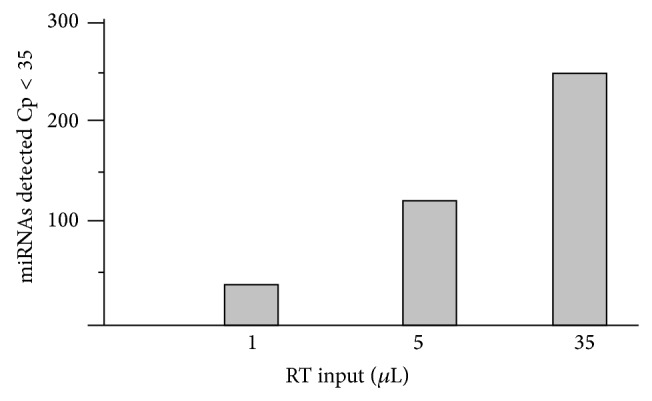
miRNA detected in three increasing mitochondrial mt-RNA inputs.

Figure 14 shows number of miRNA detected in three increasing mitochondrial mtRNA inputs. The number of assays giving a significant signal (*C*_*p*_ < 35) was 43, 123, and 251, respectively (Figure 14). The maximum number of assays giving a significant signal represents 24% of the total amount of miRNA assays tested (Table 2).

## 4. Discussion

In this study we have identified six novel human miRNA and their precursor's pre-miRNA inside the mitochondria isolated from human muscular cells. The experiments were conducted on skeletal muscular cell cultures in order to facilitate the enrichment of mitochondrial population using a specialized method. The specific detection of pre-miRNA and miRNA was performed by quantitative Northern analysis and further matured miRNA was significantly detected by RT-PCR in mitochondria RNA extract. Moreover cytoplasmic contamination of the mitochondrial fraction was carefully addressed in this study. The mitochondrial fraction was isolated with a very selective extraction method consisting of super paramagnetic microbeads conjugated to an outer membrane protein antibody. This method was validated by comparison with two other methods, differential centrifugation and ultracentrifugation, on Percoll gradient [42]. Magnetic cell sorting (MACS) method was found to be better than differential centrifugation method but in case of Percoll gradient both methods were quite similar. We have also observed that very less contaminants such as endoplasmic reticulum and nucleus were detected by antibodies in the mitochondria fraction compared to the differential centrifugation method.

The mitochondrial fraction was assessed by various methods at the protein, mRNA, DNA, and ultrastructure level. We found very low cytosolic mRNA contamination and high mt-RNA enrichment at protein level. In Western blotting analysis two specific proteins from cytosol were poorly detected in mitochondrial fraction whereas two specific mitochondrial proteins were highly enriched. Mitochondrial RNA importation is present in many organisms, including protozoans, plants, fungi, yeasts, and animals. In plants tRNA importation is depicted by voltage dependent anion channel VDAC [43]. tRNA importation in mitochondria is a known process and it might also play a role for the mitochondrial miRNA importation [21, 44]. The Argonaute Ago2 is one of the most important proteins of RISC (RNA induced silencing complex) and it could be involved in the final step of the mitochondrial miRNA interference process [45]. Transport of tRNA^Met^ by Argonaute Ago2 out of the mitochondria to the cytosol is also known in human [46].

The production of induced mutations for targeted inactivation of mtRNA in the mitochondria is far from being achieved. It is difficult to make direct test for identification of mt-miRNA biological functions. However we are using MT-RNR2 gene transfected cells for target identification of hsa-miR-mit3 and hsa-miR-mit4 microRNA using luciferase assay. For predicted target of six novel miRNA, we have employed an experimental RNomics approach to define complete targets including different mitochondrial diseases. Therefore, targeting mitochondrial DNA and diseased genes by means of employing different predicted tools is one of the promising and reliable approaches. As a direct example of mitochondrial target, results of the luciferase assay were used. Data showed that hsa-miR-mit3 and hsa-miR-mit4 downregulate MT-RNR2 gene expression. However the expression level of MT-RNR2 gene was equal in control cells. MT-RNR2 is a key mitochondrial gene, which encoded 16S RNA in mitochondria. It is associated with important diseases such as Alzheimer disease and Type II diabetes, so maybe these miRNA have role in human disease regulation.

The present study demonstrated existence of six novel miRNA and their precursor from human mitochondria of skeletal muscular cells. Two different datasets confirmed the presence of six novel miRNA and their precursor in the purified mitochondrial RNA extraction. Bioinformatics analysis allows us to examine the existence and evolution of these miRNA in other mammals, which will explain the possible origin during evolution. Moreover, presence of miRNA and pre-miRNA in the mitochondria also explain the existence of miRNA processing machinery either transported through the membrane of mitochondria or processed pre-miRNA is transported inside the mitochondria, which should be further investigated. Our results also raise a new question regarding the miRNA function in controlling different diseases. Finally it is interesting to observe that RT-PCR from purified mitochondria extract allows us to identify 6 potential miRNA described in the muscle cell. These miRNA have function in different diseases including myogenesis and fibrosis and oncogenic or oncogenic suppressor activities. The transcription factor p53 has been also co-localized in the mitochondria during p53 dependent apoptosis and it is putative regulator for other described roles in mitochondria. In addition p53 regulates miRNA processing machinery; thus miRNA localization in mitochondria would be related to apoptosis and involved in tumorigenesis and unbalanced transcription activity. Therefore the posttranscription silencing by the miRNA interference could be one of the explanations of the unbalanced number of mitochondria gene transcripts which are processed altogether in one long transcript and then cleaved in several independent gene transcripts.

At present our study provides no substantial evidence for a pathway that could promote the import of miRNA machinery into the cell organelles to a significant extent. The nuclear derived miRNA was found in a significant amount in mitochondria. However we cannot exclude that some of the identified nuclear proteins are imported in the mitochondria at a significant level. In summary, we present the first comprehensive analysis of human mitochondrial miRNA identification, characterization, miRNA target identification, and miRNA target network analysis. Finally, posttranscriptional regulation by RNA silencing in the mitochondria may exist if the miRNA enzymatic machinery could be imported and active in this organelle which should be further investigated. Our results pave a new avenue of research regarding the miRNA biogenesis in mitochondria and their functions in the regulation of mitochondrial genome.

## Supplementary Material

Table S1 has the information of Physico-Chemical Properties of 6 pre and mature miRNAs. Each column contains information such as %GC Content, %AU Content, Length, mol wt and Free energy of miRNAs. Table S2 has the list of predicted Human pre-microRNA and their genomic locations on mitochondrial genome. First Column Human miRNA has list of miRNA, second and third column has Start and end position of miRNAs, last fourth column has the pre-miRNA Sequences. Table S3 has the list of predicted Human mature-miRNA and their genomic location on mitochondrial genome. First column Human miRNA has list of miRNA, second and third column has start and end position of miRNAs and last fourth column has the mature miRNA sequences. Table S4 has the information of miRNA targets for miRanda analysis. First column has numbers, second has information of miRNAs targeting disease specific genes, third column has the location of genes which are targeted by miRNAs, fourth column has list of miRNAs and fifth and sixth column has start and end position of miRNAs. Table number S5 has miRNA targets for miRanda and RNA22 analysis. First column has serial numbers, second column contains information of miRNA targeting disease specific genes, third has the location of genes which are targeted by miRNAs, fourth column has list of miRNAs and fifth and sixth column has start and end position of miRNAs. Table number S6 has information of mitochondrial gene position and description. Column number one has serial numbers, second column has map locus, third has map position (np), fourth has shorthand of genes and last fifth column has description of genes.

## Figures and Tables

**Table 1:** Showing binding sites start and end positions of all predicted miRNA.

miRNA	Human		Chimp		Orangutan		Mouse		Rat		Rhesus
	Start position	End position	Start position	End position	Start position	End position	Start position	End position	Start position	End position	
hsa-miR-mit-1	6715	6735	6180	6200	6152	6172	—	—	—	—	—
hsa-miR-mit-2	8454	8472	7878	7896	—	—	—	—	—	—	—
hsa-miR-mit-3	9186	9207	8603	8624	8641	8662	—	—	—	—	—
hsa-miR-mit-4	10832	10851	10249	10268	—	—	—	—	—	—	—
hsa-miR-mit-5	5094	5115	4511	4532	—	—	—	—	—	—	—
hsa-miR-mit-6	2406	2426	1823	1843	—	—	1935	1955	1920	1940	—

**Table 2:** Mitochondrial microRNA detected in three increasing mitochondrial mt-RNA inputs (1 *µ*L, 5 *µ*L, and 35 *µ*L).

microRNA	1 *µ*L	5 *µ*L	35 *µ*L	Count
hsa-miR-mit-1	32.54	28.27	24.18	3
hsa-miR-mit-2	35.65	31.78	28.45	3
hsa-miR-mit-3	33.44	29.82	25.72	3
hsa-miR-mit-4	38.76	34.12	30.43	3
hsa-miR-mit-5	39.79	37.68	33.73	3
hsa-miR-mit-6	39.40	38.56	36.69	3
